# Alanine Uptake Is Required to Maintain *Staphylococcus aureus* Cell Envelope Stability Under Magnesium and Calcium Limitation

**DOI:** 10.3390/microorganisms14061332

**Published:** 2026-06-13

**Authors:** Tyler G. Brown, Shalee Killpack, Vinai Thomas, David L. Erickson, Eric Wilson

**Affiliations:** 1 Department of Microbiology and Molecular Biology, Brigham Young University, Provo, UT 84604, USA; brown248@byu.edu (T.G.B.); skillpack15@gmail.com (S.K.); david_erickson@byu.edu (D.L.E.); 2 Department of Pathology, Microbiology and Immunology, University of Nebraska Medical Center, Omaha, NE 68198, USA; vinai.thomas@unmc.edu

**Keywords:** *Staphylococcus aureus*, alanine metabolism, cell envelope stability

## Abstract

The cell envelope of Gram-positive bacteria is a primary target of host immune defenses and antibiotics, and its stability is influenced by environmental factors, including the availability of the divalent cations Mg^2+^ and Ca^2+^. Alanine also plays a critical role in cell envelope integrity, contributing to peptidoglycan cross-linking, D-alanine modification of teichoic acids, and protein synthesis. However, how these factors functionally interact to maintain envelope stability in *S. aureus* remains unclear. Here, we demonstrate that growth of *S. aureus* under Mg^2+^-limited and Ca^2+^-limited conditions requires increased alanine uptake mediated by the transporter AapA. Loss of AapA results in increased cell lysis and impaired growth under cation-limited conditions, and removing alanine from the growth medium phenocopies these *aapA* mutant defects. Alanine limitation increases susceptibility to the detergent Triton X-100 and the membrane-targeting antibiotic daptomycin, consistent with defects in envelope stability. Furthermore, *aapA* function contributes to bacterial fitness in insect and murine infection models. Together, these findings indicate that Mg^2+^, Ca^2+^, and alanine play overlapping roles in stabilizing the *S. aureus* cell envelope, pointing to AapA as a target that may leveraged to enhance antimicrobial efficacy.

## 1. Introduction

*Staphylococcus aureus* is a Gram-positive pathogen frequently implicated in invasive and chronic human infections. Clinical isolates often exhibit intrinsic antibiotic tolerance and a high incidence of resistance [1,2], posing a persistent challenge to treatment. Antibiotics of last resort against resistant *S. aureus*, such as vancomycin and daptomycin, target the bacterial cell envelope; in response to envelope stress caused by antibiotics or host-derived antimicrobials, *S. aureus* remodels its cell wall to enhance survival [3,4,5,6,7].

In addition to the role of L-alanine in proteinogenesis, L- and D-alanine are essential for peptidoglycan synthesis and modification of teichoic acids in the cell wall [reviewed in [8,9]]. Cellular alanine requirements can be met through *de novo* biosynthesis or uptake from the environment. The AapA transporter (SAUSA300_1642), also known as CycA, mediates efficient uptake of both D- and L-alanine, and deletion of *aapA* increases sensitivity to D-cycloserine and β-lactam antibiotics [10,11]. Although inactivation of *aapA* substantially slows alanine depletion from the culture medium [10], residual uptake indicates that AapA is not the sole alanine transporter. However, genome-wide screens suggest that *aapA* contributes to fitness under stress conditions, including abscess formation, salt stress, and daptomycin exposure [11,12,13,14]. *De novo* alanine synthesis requires D-alanine aminotransferase (Dat) and alanine racemase (Alr) [15], and previous studies suggest that *de novo* synthesis is insufficient to meet demand under certain stress conditions [11,12,15], suggesting a critical role for environmental alanine acquisition.

Envelope stabilization by the divalent cations Mg^2+^ and Ca^2+^ has been reported in some Gram-positive bacteria; these cations protect cells from lysis and antimicrobial targeting of the membrane [16,17,18,19]. However, it is unclear if this stabilizing effect applies to *S. aureus*, as excessive concentrations of these divalent cations can be lethal to *S. aureus* specifically [20]. Thus, the role of Mg^2+^ and Ca^2+^ associated with the cell envelope at physiological concentrations remains poorly defined in *S. aureus*, and the functional interplay between alanine uptake and envelope-associating divalent cations is not yet well understood.

During infection, host nutritional immunity limits the availability of essential bacterial nutrients, including the divalent cations Mg^2+^ and Ca^2+^ [21,22]. In this study, we investigated how alanine uptake contributes to bacterial survival under cation-limited conditions. We show that Mg^2+^ and Ca^2+^ limitation increases the cellular requirement for alanine and that this demand cannot be met by biosynthesis alone. Instead, AapA-mediated uptake is required to maintain growth and prevent lysis in some strains of *S. aureus*. Furthermore, *in vivo* infection models confirm the importance of AapA for fitness in a host environment.

## 2. Materials and Methods

### 2.1. Bacterial Growth Conditions

*Staphylococcus aureus* strains were routinely cultured on tryptic soy agar (TSA) medium at 37 °C. All experiments measuring growth began with overnight culture in tryptic soy broth (TSB). A 1:50 dilution of the overnight culture was then grown to mid-exponential phase, at which point cells were pelleted and resuspended in phosphate-buffered saline (PBS; 8 g/L NaCl, 0.2 g/L KCl, 1.44 Na_2_HPO_4_, 0.24 KH_2_PO_4_). These cells were then inoculated into experimental media to a final optical density (OD_600_) of 0.01 in a 96-well plate. OD_600_ was measured hourly using a BMG SpectroStar Nano plate reader with shaking at 37 °C (BMG LABTECH GmbH, Ortenberg, Germany). All experimental growth tests were carried out in a chemically defined medium adapted from Hussain et al. [23] and Sebulsky et al. [24] and detailed in the Supplemental Materials. The basal chemically defined medium is prepared with low Mg^2+^/Ca^2+^, defined as 5 mg/L MgSO_4_ (41 µM Mg^2+^) and 5 mg/L CaCl_2_-2H_2_O (34 µM Ca^2+^). All amino acids (Sigma-Aldrich, St. Louis, MO, USA) excluding alanine were added to this basal medium. Where appropriate, alanine was added to a specified concentration, and an additional 2 mM Mg^2+^ and/or 2 mM Ca^2+^ was added to create high Mg^2+^/Ca^2+^ conditions.

### 2.2. S. aureus Strain Preparation

All strains used in this study are detailed in Table 1. Transposon mutants (*aapA*::tn and *alr*::tn) were provided by the Nebraska Transposon Mutant Library [25]. Plasmids were maintained (as in the *aapA* complemented strain and as appropriate during allelic exchange) using 10 µg/mL chloramphenicol in overnight cultures. For allelic exchange to create a *dat* deletion mutant, the flanking regions of *dat* were amplified by PCR (5′-CATAGAGGTACCCAACCTCGCCTGTTTCTATG, 5′-TTTATTCATTATGCGTGGAGGAATAATATAATTCTTTCATCATATTTTTAGATTAAATT for amplification of downstream flanking region and 5′-ATATTATTCCTCCACGCATAATGAATAAA, CATAGAGGTACCGGATCCATCTATCGGTGTGAATG for upstream flanking region). PCR products were then stitched together using overlap extension PCR. The PCR product and pIMAY-Z plasmid [26] were digested using KpnI, and the plasmid was then phosphatase treated, and digested products were then ligated using T4 DNA ligase and transformed into chemically competent *E. coli* strain IM08B [26]. The plasmid was isolated from IM08B and transformed by electroporation into *S. aureus* strain JE2. The process of allelic exchange was then performed as detailed by Monk et al. [26]. For complementation of *aapA*, the *aapA* gene and promoter were amplified by PCR (5′-ACAGGAGCTCGATAAATTTCCCCTTTACTGTTTCTTTATG and 5′-ATAGGGTACCAGCTCAACGAGCTGTACATTAT). The PCR product and pRMC2 plasmid [27] were digested using the SacI and KpnI enzymes, and ligated together. The ligation product was transformed into IM08B, PCR verified, then isolated and transformed by electroporation into the *aapA* mutant.

Diverse isolates tested for growth in low Mg^2+^ and Ca^2+^ with no alanine included clinical human-derived strains isolated by others (see Table S1), as well as bovine mastitis strains (UH5 and IJ4) isolated from infected dairy cow milk as part of this study. DNA isolated from UH5 was Illumina- and Nanopore-sequenced by SeqCenter (Pittsburgh, PA, USA) services. This resulted in contiguous chromosome and plasmid sequences (NCBI accession ID: SAMN57330621). DNA isolated from IJ4 was Illumina-sequenced, also by SeqCenter (NCBI accession ID: SAMN57331713).

### 2.3. Supernatant eDNA Quantification Using Propidium Iodide

As an indirect measure of cell lysis, overnight cultures were first sub-cultured in TSB, washed, and then inoculated into defined medium at an OD_600_ of 0.1 in a volume of 2 mL and incubated for 4 h with shaking at 37 °C. Cells were then pelleted by centrifugation, and the supernatant collected. Propidium iodide was added to the supernatant at a final concentration of 0.5 µg/mL. This was then vortexed thoroughly and incubated at room temperature in the dark for 10 min, followed by measurement of propidium iodide fluorescence in a 96-well plate at 490/640 nm excitation/emission wavelengths.

### 2.4. Triton X-100 Sensitivity Assay

As a test of cell envelope integrity, strains were grown overnight in TSB, then sub-cultured in TSB for 2 h. These cells were then washed in PBS and resuspended in fresh defined medium with the specified alanine/cation additives, including 0.05% Triton X-100. These cells were then transferred to a 96-well plate and incubated with shaking at 37 °C. OD_600_ was measured hourly using a BMG SpectroStar Nano plate reader and reported as a percentage of the OD_600_ measurement at the first hour after inoculation (BMG LABTECH GmbH, Ortenberg, Germany).

### 2.5. Daptomycin Susceptibility Testing

Susceptibility to daptomycin (MedChemExpress, Monmouth Junction, NJ, USA) was tested according to our standard growth assay described above. The specified concentration of daptomycin was added to cells at an OD_600_ of 0.01 and growth was measured hourly in defined medium containing 2 mM Ca^2+^ and 0.04 mM Mg^2+^ with either 0.1 or 1 mM L-alanine.

### 2.6. Infection of Galleria mellonella

*G. mellonella* were infected by injection of 10^5^ colony forming units (CFUs) of either wild-type JE2 or the *aapA* mutant prepared by subculturing overnight cultures in TSB, resuspending and diluting in PBS, and then injecting 10 µL at the third left proleg (*n* = 10 per group in 3 separate experiments). The infected larvae were then incubated at 37 °C and death of larvae was assessed at 24-h intervals. Control larvae were injected with PBS alone.

### 2.7. Infection of RAW 264.7 Cells

The mouse macrophage-derived RAW 264.7 cells were grown in 50/50 DMEM/RPMI with 10% FBS and antibiotics. The 6-well plates were seeded and allowed to incubate for 24 h, and 70–80% confluency was confirmed by visual inspection. Fresh medium without antibiotic was added to the RAW 264.7 cells, and they were then infected with *S. aureus* at an MOI of ~30, with equal portions of wild-type JE2 and the *aapA* mutant (confirmed by CFU counts). Synchronized phagocytosis was then accomplished by centrifugation (5 min at 300 RPM) and the cells were allowed to incubate for 30 min. Gentamicin (100 µg/mL) was then added to kill extracellular bacteria. The gentamicin was removed after 30 min and fresh medium added. As a control, treatment of the inoculum suspended in DMEM (without exposure to RAW cells) using this gentamicin treatment method was confirmed to eliminate detectable CFUs. The bacteria from two wells were immediately collected using 0.01% Triton X-100 and plated to determine the intracellular CFUs at this point. Cells from the remaining four wells were collected in a similar manner 5 h after gentamicin treatment. Plating was done on TSA and TSA + 5 µg/mL erythromycin to differentiate recovered wild-type cells versus *aapA*::tn cells (which are erythromycin resistant). The competition index (CI) was calculated for the *aapA* mutant relative to the wild-type strain at 0 h (immediately after gentamicin treatment) and 5 h (5 h post-gentamicin treatment). The CI of *aapA*::tn = (CFUs of *aapA*::tn in experimental condition/CFUs of *aapA*::tn in inoculum)/(CFUs of JE2 Wt in experimental condition/CFUs of JE2 Wt in inoculum). This was performed 4 times in independent experiments.

### 2.8. Subcutaneous Infection of Mice

BALB/cJ mice were infected subcutaneously with an average of 5.5 × 10^7^ CFUs/mL with 1:1 JE2:*aapA*::tn strains in two separate experiments. In total, 4 male and 4 female mice were infected (*n* = 4 mice per experiment). Infected mice were euthanized after 48 h and infected tissue and the spleen harvested. Harvested samples were homogenized in 1 mL PBS and plated for CFUs. Plating was done on TSA and TSA + 5 µg/mL erythromycin to differentiate recovered wild type versus *aapA*::tn, as erythromycin resistance is encoded within the transposon inserted into *aapA*. The same JE2:*aapA*::tn mix used for injections was also grown for 24 h in TSB and plated as a control.

### 2.9. Statistical Analysis

Statistical analyses were performed using GraphPad Prism 10 software (GraphPad Software, San Diego, CA, USA). Statistically significant differences in endpoint growth assays were assessed using a two-way ANOVA, with the exception of multi-strain comparisons, which were analyzed using a one-way ANOVA. *In vivo* competition assays were analyzed using a one-sample *t*-test with a theoretical mean of 1. Other infections were analyzed using a two-sample *t*-test. Growth curve experiments are reported as the mean of three separate experiments, with error bars showing the Standard Error of the Mean (SEM) between experiments.

## 3. Results

### 3.1. Alanine Starvation of Staphylococcus aureus Results in Defective Growth and Increased Lysis When Mg^2+^ and Ca^2+^ Are Limited

In *S. aureus*, growing evidence indicates that alanine uptake by the alanine uptake transporter AapA (SAUSA300_1642) is necessary to survive envelope-targeting antibiotics, suggesting a role in envelope stress tolerance [10,11]. Mg^2+^ and Ca^2+^ are also likely to influence cell envelope stability, yet their contributions to cell envelope physiology remain poorly understood in *S. aureus*. To investigate the relationship between divalent cation availability and alanine transport, experiments were conducted using the USA300-LAC derivative strain JE2 in a chemically defined medium. In this medium, 0.04 mM and 2 mM Mg^2+^/Ca^2+^ are defined as low and high concentrations, respectively.

**Figure 1:**
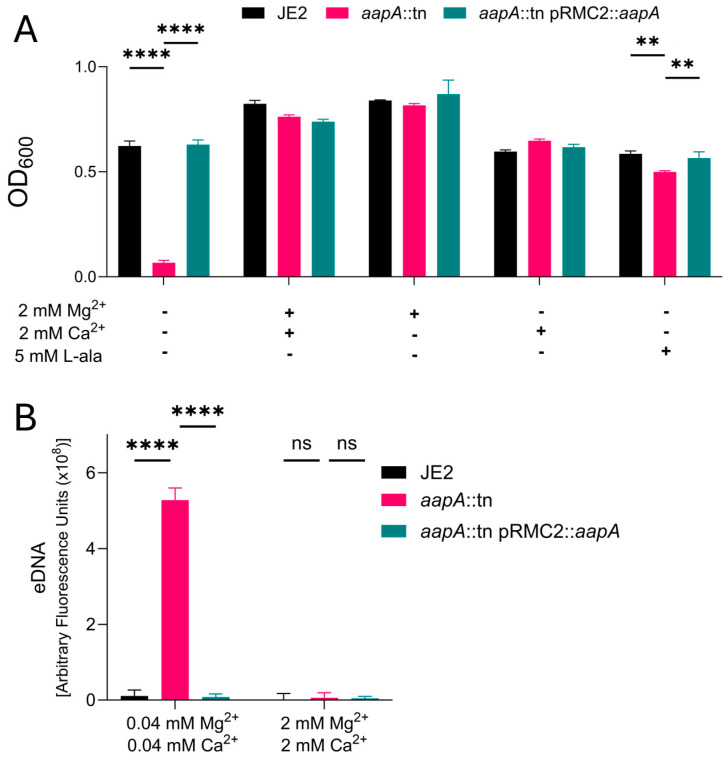
Alanine uptake by the AapA transporter is necessary for *S. aureus* (strain JE2) growth and protection from cell lysis when cultured in minimal concentrations of Mg^2+^ and Ca^2+^. (**A**) Optical density following 10-h incubation in chemically defined media containing 1 mM L-alanine and 0.04 mM each of Mg^2+^ and Ca^2+^. These basal concentrations are designated with (−), and the medium was also supplemented with additional alanine, Mg^2+^, or Ca^2+^, as indicated in the figure with (+). (**B**) Relative levels of *S. aureus* eDNA in culture supernatants following incubation in defined medium with low Mg^2+^ and Ca^2+^ or supplemented with 2 mM Mg^2+^ and Ca^2+^. Data are displayed as the mean +/− SEM of 3 biological replicates containing 3 technical replicates per experiment. Statistical significance was calculated using a two-way ANOVA (** = *p*-value < 0.005; **** = *p*-value < 0.0005).

Growth of *S. aureus* JE2 and an *aapA* transposon mutant in the same strain (*aapA*::tn) was measured in a chemically defined medium containing 1 mM L-alanine and low Mg^2+^/Ca^2+^. In these conditions, *aapA*::tn exhibited a significant growth defect compared to the wild type. However, the *aapA* mutant grew similar to the wild-type strain when the medium was supplemented with an additional 5 mM of L-alanine, high Mg^2+^, or high Ca^2+^. Complementation of the mutant with a functional copy of *aapA* also restored normal growth (Figure 1A). Given the role of the divalent cations Mg^2+^ and Ca^2+^ in cell envelope stability in other species [16,17,18,19], we hypothesized that the growth defect observed in the *aapA* mutant when cultured in low Mg^2+^/Ca^2+^ conditions was due to increased cell lysis. As a proxy for cell lysis, extracellular DNA (eDNA) was measured in culture supernatants. Supernatant eDNA from *aapA*::tn was significantly increased relative to that of the wild-type strain following growth in low Mg^2+^/Ca^2+^ conditions. In contrast, after growth in medium containing high Mg^2+^/Ca^2+^, eDNA levels in the *aapA* mutant and wild-type JE2 supernatants were equally low (Figure 1B). We interpret this to mean that alanine uptake prevents cell lysis in low Mg^2+^/Ca^2+^ conditions, suggesting a role for alanine uptake or Mg^2+^ and Ca^2+^ in cell envelope maintenance.

### 3.2. Mg^2+^/Ca^2+^ Depletion Increases Alanine Demand in S. aureus

**Figure 2:**
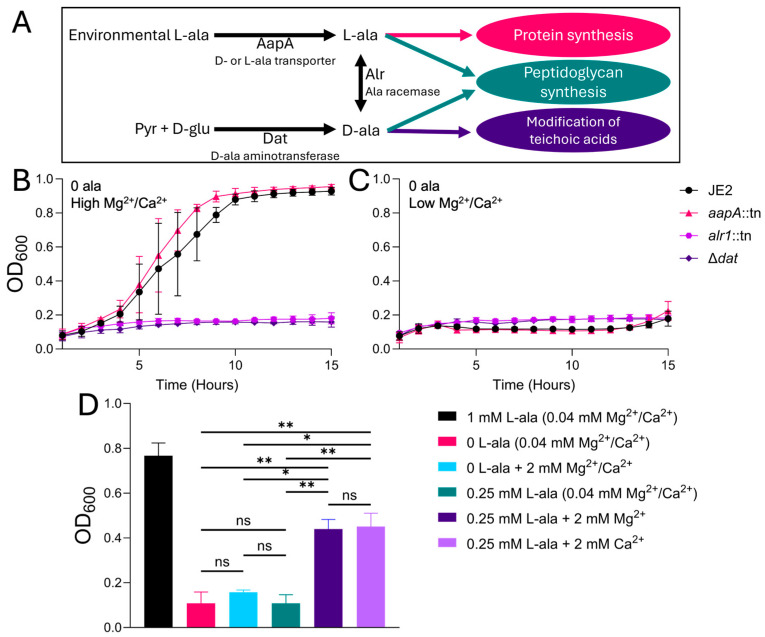
Growth of *S. aureus* lacking either exogenous alanine or endogenous alanine synthesis demonstrates an increased alanine requirement under Mg^2+^ and Ca^2+^ limitation. (**A**) A depiction of alanine metabolism in Staphylococcus aureus. (**B**) Growth of wild-type JE2, the alanine transporter gene *aapA* transposon mutant (*aapA*::tn), the alanine racemase gene *alr* transposon mutant (*alr*::tn), or a strain with a clean deletion of the alanine synthesis gene *dat* (∆*dat*) in defined medium containing no alanine and 2 mM Mg^2+^/Ca^2+^ or (**C**) no alanine and low (0.04 mM each) Mg^2+^/Ca^2+^. (**D**) Growth at 10 h of the alanine-synthesis defective dat deletion mutant strain with no exogenous alanine (0 L-ala) or 0.25 mM L-ala in defined medium containing low Mg^2+^ and Ca^2+^ (0.04 mM each) or 2 mM Mg^2+^ and Ca^2+^ supplemented. Data are displayed as the mean +/− SEM of 3 biological replicates containing 3 technical replicates per experiment. Statistical significance was calculated using a two-way ANOVA. (* = *p*-value < 0.05; ** = *p*-value < 0.005).

The results above suggest overlapping roles for Mg^2+^/Ca^2+^ and alanine in maintaining cell envelope stability and confirm that alanine uptake via AapA somehow compensates for environmental depletion of these divalent cations. However, *S. aureus* can supply its own alanine through *de novo* synthesis. Dat synthesizes D-alanine from glutamate, and Alr catalyzes interconversion between D- and L-alanine (Figure 2A) [15]. This raises the question as to why Dat/Alr-dependent *de novo* synthesis is unable to support growth of the *aapA* mutant in low Mg^2+^/Ca^2+^. We investigated the possibility that alanine demand increases as Mg^2+^/Ca^2+^ concentrations decrease, and that this increased demand cannot be met by Dat/Alr biosynthesis. To address this possibility, we obtained *dat* and *alr* mutants (Δ*dat* and alr::tn, respectively) and confirmed their inability to grow in the absence of exogenous alanine (Figure 2B). Under the same conditions, JE2 and *aapA*::tn exhibit robust growth, as would be expected. However, when alanine is absent and Mg^2+^/Ca^2+^ are low (Figure 2C), all four strains fail to grow. This shows that Dat and Alr can supply adequate D- and L-alanine for growth only when Mg^2+^/Ca^2+^ concentrations are sufficiently high. This finding rules out the notion that AapA-dependent uptake is somehow inhibited by low Mg^2+^/Ca^2+^ concentrations. If this were the case, growth of the wild type in the absence of exogenous alanine (where AapA is superfluous) would not be inhibited in low Mg^2+^/Ca^2+^. We went on to assess how much exogenous L-alanine is required to support growth of the Δ*dat* strain in low Mg^2+^/Ca^2+^ (Figure 2D), finding that 1 mM L-alanine is sufficient to restore robust growth, while 0.25 mM L-alanine is not sufficient. However, when Mg^2+^ or Ca^2+^ levels are elevated to 2 mM, 0.25 mM L-alanine partially restores growth to the Δ*dat* strain. This finding allows us to reject the notion that Dat biosynthetic activity is somehow inhibited by low Mg^2+^/Ca^2+^; if this were the case, the amount of exogenous alanine required for growth of a *dat* mutant would not vary as a function of Mg^2+^/Ca^2+^ concentration. Instead, our results point to low Mg^2+^/Ca^2+^ imposing a higher cellular demand for alanine, independent of its source.

### 3.3. Exogenous Alanine Starvation Results in Increased Sensitivity to Membrane Destabilization

**Figure 3:**
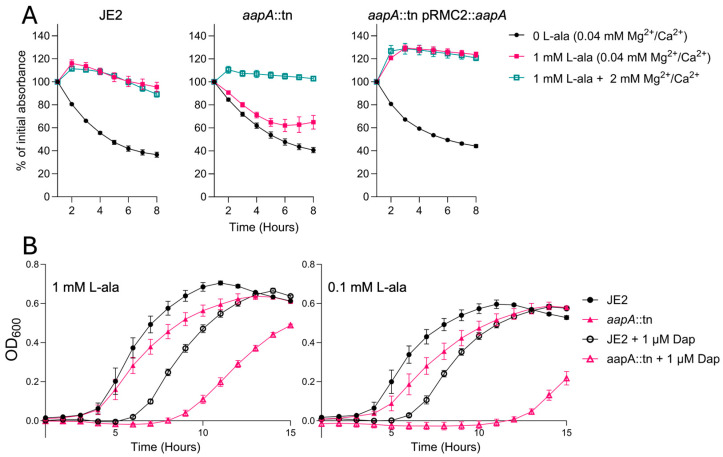
Tolerance of *S. aureus* to membrane perturbation by Triton X-100 in low Mg^2+^ and Ca^2+^ or daptomycin treatment requires efficient alanine uptake or 2 mM Mg^2+^/Ca^2+^. (**A**) Lysis in the presence of 0.05% Triton X-100 of the wild-type JE2, the alanine transporter mutant (*aapA*::tn), or the complemented *aapA* mutant (*aapA*::tn pRMC2::*aapA*) in defined medium containing 0 alanine and low (0.04 mM each) Mg^2+^ and Ca^2+^ or with the addition of 1 mM L-alanine in low Mg^2+^ and Ca^2+^, or 1 mM L-alanine and 2 mM Mg^2+^ and Ca^2+^. (**B**) Growth of wild-type JE2 or alanine transporter mutant *aapA*::tn in defined medium containing 2 mM Ca^2+^ and 0.1 or 1 mM L-alanine with or without 1 µM Daptomycin. Data are displayed as the mean +/− SEM of 3 biological replicates containing 3 technical replicates per experiment.

We hypothesized that the increase in alanine demand in low Mg^2+^/Ca^2+^ is attributable to reinforcing cell wall components such as peptidoglycan or teichoic acids (both of which require alanine). It has been shown previously that *S. aureus* cell wall defects potentiate cell lysis by the membrane-destabilizing detergent Triton X-100 [31]. Thus, we expected alanine-starved cells to be Triton sensitive. To test Triton sensitivity, bacteria were collected during exponential growth in TSB, washed, and then suspended in media containing 0.05% Triton X-100 and specified amounts of Mg^2+^/Ca^2+^ and alanine (see Figure 3A). Optical density was then used to measure lysis over 8 h. Lysis of wild-type bacteria occurs in low Mg^2+^/Ca^2+^ when alanine is absent from the medium, but is prevented by supplementation of 1 mM L-alanine. For the *aapA* mutant, however, lysis occurs in either condition. We conclude from these results that *S. aureus* requires exogenous alanine to reinforce the cell wall in low Mg^2+^/Ca^2+^ conditions, subsequently preventing detergent-induced lysis.

The antibiotic daptomycin targets the membrane of Gram-positive bacteria through a Ca^2+^-dependent mechanism, causing membrane depolarization and lysis [32,33]. In response to sublethal daptomycin exposure, *S. aureus* stabilizes its cell wall through increased peptidoglycan synthesis and D-alanine modification of teichoic acids [6,34,35]. Given our evidence that these processes may be compromised by exogenous alanine starvation, we expected exogenous alanine uptake to influence daptomycin sensitivity. Daptomycin sensitivity was measured by comparing the growth of wild-type and *aapA* mutant bacteria upon exposure to 1 µM daptomycin in media containing 1.0 or 0.1 mM L-alanine in low Mg^2+^. Daptomycin treatment moderately extended the lag phase for the wild-type strain, with equal growth delays in 1.0 and 0.1 mM L-alanine conditions. For the *aapA* mutant, the lag phase exceeded that of the wild type by approximately 3 h in 1 mM L-alanine; in 0.1 mM L-alanine, the lag phase exceeded that of the wild type by approximately 7 h (Figure 3B). Importantly, these experiments were performed in the presence of high Ca^2+^ (required for daptomycin activity), indicating that alanine uptake is critical to tolerate daptomycin-induced membrane stress even when divalent cations are available.

### 3.4. The Requirement for Exogenous Alanine Varies Among S. aureus Strains

**Figure 4:**
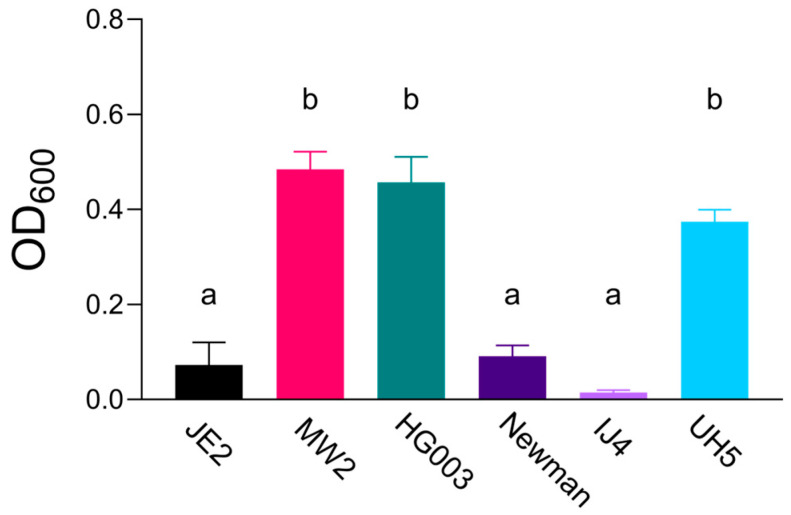
Responses of *S. aureus* to alanine, Mg^2+^, and Ca^2+^ depletion varies between strains. *S. aureus* strains were grown in defined medium containing low (0.04 mM each) Mg^2+^/Ca^2+^ and no alanine and growth measured at 15 h. Strains include the USA300 lineage strain JE2 (ST8), human isolate MW2 (ST1), lab strain HG003 (ST8), human isolate Newman (ST8), bovine mastitis isolate IJ4 (ST151) and bovine mastitis isolate UH5 (ST2187). Letters denote statistically significant differences (*p*-value < 0.05 in a one-way ANOVA) between strains designated “a” and those designated “b”. Data are displayed as the mean +/− SEM of 2 biological replicates containing 3 technical replicates per experiment.

We next sought to determine if the increased demand for alanine uptake in low Mg^2+^/Ca^2+^ is shared across *S. aureus* strains of diverse sequence types (ST) and methicillin resistance traits (MSSA/MRSA). Growth of tested strains in low Mg^2+^/Ca^2+^ containing no alanine varied substantially, with JE2 (ST8/MRSA), Newman (ST254/MSSA), and IJ4 (ST151/MSSA) strains exhibiting negligible growth, whereas MW2 (ST1/MRSA), HG003 (ST8/MSSA), and UH5 (ST2187/MSSA) strains were capable of growth (Figure 4). These results indicate that individual strains differ significantly in their need for exogenous alanine uptake when cultured in low Mg^2+^/Ca^2+^ conditions.

### 3.5. AapA Contributes to Bacterial Survival During Infection

**Figure 5:**
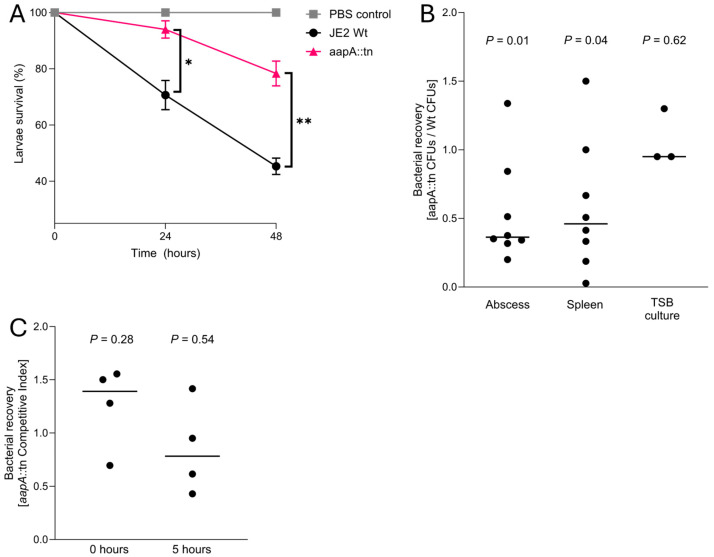
Mutation of *aapA* attenuates the infective capacity of *S. aureus*. (**A**) *G. mellonella* larvae survival after inoculation with 10^5^ CFUs of wild-type JE2 or *aapA*::tn and incubation at 37 °C for 48 h, with survival assessed every 24 h. Injections were performed in groups of at least 10 larvae in 3 separate experiments. Significance was calculated using a two-way ANOVA. (**B**) Ratio of *aapA*::tn to wild type recovered after 48 h from a murine co-infection with equal quantities of both strains introduced by subcutaneous injection. Two experiments were performed (*n* = 4 per experiment). Significance for mouse infection was calculated using a theoretical mean of 1 in a one-sample *t*-test. (**C**) Survival of *S. aureus* after synchronized phagocytosis by RAW cells assessed immediately after gentamicin treatment of RAW cells (0 h) and 5 h after gentamicin treatment (5 h). RAW cell testing was performed in quadruplicate in four separate experiments. The competitive index was calculated using the CFUs either immediately after gentamicin treatment (T0) or 5 h after gentamicin treatment (T5) relative to the inoculum. Immediately after gentamicin treatment, a non-significant increase in phagocytosed *aapA*::tn cells relative to wild-type cells was observed, as well as a non-significant decrease in viability after 5 h. Significance was calculated using a theoretical mean of 1 in a one-sample *t*-test. (* = *p*-value < 0.05; ** = *p*-value < 0.005).

*Galleria mellonella* insect larvae have been used to measure intrinsic virulence properties of *S. aureus* [36]. The insect immune system controls bacterial infection through a combination of innate immune factors, including phagocytic cells and antimicrobial peptides. Upon *G. mellonella* infection, we observed that *aapA*::tn exhibited significantly reduced virulence compared to wild-type bacteria, as measured by survival of larvae (Figure 5A). Next, we measured bacterial survival in a murine subcutaneous infection model. In competition assays between wild-type and *aapA*::tn strains, significantly fewer *aapA*::tn CFUs were recovered from abscesses relative to the wild-type strain. Spleens harvested from the co-infected mice also contained fewer *aapA*::tn than wild-type bacteria, although the ratio of recovered CFUs was more variable than in abscesses (Figure 5B).

Assays were next designed to specifically assess intracellular survival of wild-type and *aapA*::tn bacteria following phagocytosis by murine macrophages. Gentamicin protection assays were performed by adding equal numbers of mutant and wild-type bacteria to the mouse macrophage cell line RAW 264.7 [37]. Equivalent numbers of wild-type and *aapA*::tn bacteria were recovered immediately following phagocytosis and gentamicin treatment, indicating similar invasion/phagocytosis efficiencies of both strains. Following five hours of incubation within these phagocytic cells, plating and enumeration of surviving bacteria showed a non-significant trend toward increased survival of wild-type bacteria (Figure 5C).

## 4. Discussion

Maintaining cell envelope stability is essential for bacterial growth and survival, with peptidoglycan providing the primary structural support in Gram-positive bacteria. Alanine is required for pentapeptide stem formation and cross-linking, while the divalent cations Mg^2+^ and Ca^2+^ associate with the envelope through electrostatic interactions with various envelope constituents [16]. Although these roles are well established, their functional interplay in supporting cell envelope stability has not been well defined. Here, we demonstrate that under cation-limited conditions, some strains of *Staphylococcus aureus* exhibit an increased dependence on AapA-mediated alanine uptake to maintain cell envelope integrity. Disruption of alanine acquisition in these conditions results in increased cell lysis *in vitro* and reduces fitness in infection models, linking nutrient availability to cell envelope stability.

Our findings support a model in which depletion of Mg^2+^ and Ca^2+^ shifts the burden of envelope stabilization toward alanine-dependent processes, such as peptidoglycan and teichoic acid modification. D-alanine modification of the negatively charged groups of teichoic acid neutralizes a major source of negative charge in the cell wall, and may decrease the need for ionic bridging accomplished by envelope-associated Mg^2+^ and Ca^2+^. Peptidoglycan thickening also requires alanine, and has been shown to improve tolerance of *S. aureus* to envelope stress [1,5]. Under conditions that may require these processes, such as exposure to envelope-targeting antibiotics or detergents, *de novo* alanine synthesis appears insufficient to meet alanine demand, making alanine uptake via AapA critical for growth and fitness. A previous study of *S. aureus* strain MW2 reported deficient D-alanine modification of teichoic acids in *aapA* mutant cells [11], an essential process for tolerating envelope stress [4,38]. Another study in strain JE2 found modest changes in peptidoglycan cross-link composition between the wild type and the *aapA* mutant [10]. Based on our findings, we would expect the differences in cell envelope composition between the two strains to be amplified upon experiencing a secondary stress, such as divalent cation depletion.

The variability in growth observed across *S. aureus* strains cultured under conditions of alanine and cation limitation suggests the presence of compensatory mechanisms for cell wall stabilization. These differences may reflect variation in alanine biosynthetic capacity, cation-responsive regulatory networks [39,40], or altered peptidoglycan cross-linking or teichoic acid modification [5]. We consider it most likely that all strains experience an increased demand for alanine upon divalent cation depletion, but some are better equipped to upregulate *de novo* biosynthesis.

Host nutritional immunity is typically associated with sequestration of trace metals, such as Mn^2+^ and Fe^2+^ [41,42,43]. However, additional mechanisms of nutritional immunity may target more abundant divalent cations, such as Mg^2+^ and Ca^2+^. For example, the host divalent metal transporter NRAMP1 depletes Mg^2+^ from the phagosome [22], and Ca^2+^ efflux from the neutrophil phagosome has also been documented [21]. Thus, we predicted that the *aapA* mutant may be more susceptible to the harsh environment inside phagocytic cells due to Mg^2+^/Ca^2+^ depletion and other cell envelope stressors. Consistent with this, we observe that the *aapA* mutant is less virulent than the wild type in an insect infection model and less capable of survival in a murine infection model. However, our results did not indicate a statistically significant defect in proliferation/survival after five hours of the *aapA* mutant in a macrophage cell line. This may be because intracellular *S. aureus* manipulates Ca^2+^ inside of host cells—a phenomenon that has been observed previously [44]. Alternatively, it could be that peptides available in host cells provide a sufficient supply of alanine.

Previous studies suggest AapA contributes to bacterial fitness in some host environments but not others. For example, genome-wide screens have indicated that AapA plays a role in *S. aureus* strain HG003 for survival in abscesses, and in strain TM283 (nearly isogenic to JE2 used in this study) under salt stress [12,13]. Conversely, an *aapA* mutant in strain JE2 showed minimal or no fitness defects in a bloodstream infection model [10]. These differences may reflect variation in nutrient and cation availability across host niches.

Disruption of AapA increases the sensitivity of *S. aureus* to the membrane targeting antibiotic daptomycin, as shown in this work with strain JE2, and by others with strain MW2 [11]; this is also evidenced by transposon insertion screens performed by others in various strains, including TM283, MW2, HG003 and others [14]. The *aapA* mutant also has increased sensitivity to other cell wall-targeting antibiotics, including β-lactams [10,11]. It seems likely that alanine starvation and cell wall-targeting agents act additively, with each compromising cell wall cross-linking by distinct mechanisms. Under these conditions of compromised cell wall synthesis, we have shown that *S. aureus* becomes increasingly dependent on envelope stabilization by Mg^2+^ and Ca^2+^. Links between cation chelation and envelope-targeting antibiotic susceptibility have been shown [45], and are further supported by the findings of this study.

In summary, this work identifies alanine uptake as an important determinant of cell envelope stability in *S. aureus* under conditions of divalent cation limitation. Within the context of host nutritional immunity, where Mg^2+^ and Ca^2+^ availability is restricted, alanine acquisition becomes increasingly important for maintaining cell wall integrity and preventing lysis. This conditional dependence is supported by *in vivo* observations, in which disruption of AapA-mediated alanine acquisition reduces bacterial fitness in multiple infection models. Together, these findings link nutrient limitation to cell wall homeostasis and support growing evidence that alanine uptake represents a vulnerability that could be leveraged to enhance antimicrobial strategies.

## Figures and Tables

**Table 1:** Strains used in this study.

Strain Name	Strain Specifics	Source	Additional Information
JE2	USA300-LAC derivative strain with antibiotic resistance and some restriction systems removed	Nebraska Transposon Mutant Library [25]	-
*aapA*::tn	*Bursa aurealis* transposon insertion inactivating the *aapA* gene of strain JE2	Nebraska Transposon Mutant Library [25]	See references [10,11]
*alr*::tn	*Bursa aurealis* transposon insertion inactivating the *alr* gene of strain JE2	Nebraska Transposon Mutant Library [25]	See references [11,15]
∆*dat*	In-frame deletion of *dat* gene of strain JE2	This study, created by allelic exchange according to the method of Monk et al. [26]	See references [11,15]
IM08B	Engineered *E. coli* strain that modified plasmid DNA for *S. aureus* CC8 transformation at high efficiency	Monk et al. [26]	-
MW2	USA400 lineage strain, originally isolated from septic human patient	Baba et al. [28]	-
HG003	Genetically modified derivative of strain NCTC8325, originally isolated from septic human patient	Sassi et al. [29]	-
Newman	Isolated in 1952 from invasive human infection	Duthie et al. [30]	-
IJ4	Isolated from milk of dairy cow with clinical mastitis	This study	-
UH5	Isolated from milk of dairy cow with clinical mastitis	This study	-

## Data Availability

The original contributions presented in this study are included in the article/Supplementary Materials. Further inquiries can be directed to the corresponding author.

## Supplementary Materials

The following supporting information can be downloaded at https://www.mdpi.com/article/10.3390/microorganisms14061332/s1, Table S1: Strains used in this study.
