# Supplementary material for: Alanine Uptake Is Required to Maintain Staphylococcus aureus Cell Envelope Stability Under Magnesium and Calcium Limitation
Source: Microorganisms. 2026 Jun 13;14(6):1332. doi: 10.3390/microorganisms14061332 (PMC13304347; doi:10.3390/microorganisms14061332)
Supplement: Supplementary file 1 [file microorganisms-14-01332-s001.zip › microorganisms-4346436-supplementary.pdf]

Supplemental information

Alanine uptake is required to maintain *Staphylococcus aureus* cell wall stability under magnesium and calcium limitation

Tyler Brown, Shalee Killpack, Vinai Thomas, David Erickson, Eric Wilson

Table S1. Defined medium formulation.

Component	Concentration (g/L)
MOPS	20.9 (Adjust pH to 7.4 using NaOH).
Glucose	2
NH_4SO_4	0.2
NaCl	5.8
KCl	3.7
NH_4Cl	1.1
Na_2SO_4	0.142
KH_2PO_4	0.272
L-aspartic acid	0.150
L-alanine	0*
L-arginine	0.1
L-cysteine	0.05
Glycine	0.1
L-glutamic acid	0.15
L-Histidine	0.1
L-Isoleucine	0.15
L-Lysine	0.1
L-Leucine	0.15
L-Methionine	0.1
L-Phenylalanine	0.1
L-Proline	0.15
L-Serine	0.1
L-Threonine	0.15
Tryptophan	0.1
Tyrosine	0.1
Valine	0.15
**Trace component**	**Concentration (mg/L)**
$MgSO_4$	5*
$CaCl_2$-$2H_2O$	5*
$MnCl_2$	0.01
$ZnSO_4$	2.5
Biotin	0.1
Nicotinic acid	2
D-pantothenic acid	2
Pyridoxal HCl	4
Pyridoxamine 2HCl	4
Riboflavin	2
Thiamine HCl	2
Adenine sulfate	20
Guanine sulfate	20

*This concentration is added to the basal medium, and additional supplementation is performed as required
